# S102. CANNABIS MAY PROTECT AGAINST CERTAIN DISORDERS OF THE DIGESTIVE ORGANS IN PATIENTS WITH SCHIZOPHRENIA BUT NOT IN HEALTHY CONTROLS

**DOI:** 10.1093/schbul/sby018.889

**Published:** 2018-04-01

**Authors:** Julie Aamand Olesen, Christine Merrild Posselt, Merete Nordentoft, Carsten Hjorthoej

**Affiliations:** 1 Copenhagen University Hospital, Mental Health Center Copenhagen; 2 The Parker Institute; 3 Mental Health Centre Copenhagen, The Lundbeck Foundation Initiative for Integrative Psychiatric Research (iPSYCH)

## Abstract

**Background:**

Cannabis use disorder increases both overall mortality and several cause-specific mortalities. However, an unexpected finding has been that, in patients with schizophrenia, cannabis use disorders were associated with a decreased risk of death from causes related to the digestive organs. Further indications of potentially beneficial effects of cannabis were supported by a 2016 systematic review showing protective effects of cannabis in patients with established gastrointestinal disorders. Further, schizophrenia, either in itself or through the use of antipsychotics, may interact with cannabis in various organs, potentially leading to different associations. For these reasons, we aimed to investigate the associations between cannabis and later development of disorders of the digestive organs, both in patients with schizophrenia and in healthy controls.

**Methods:**

We used the nationwide Danish registers in a prospective cohort study. All individuals born since 1955 in Denmark and diagnosed with schizophrenia were included, and matched to approximately ten controls on age and sex to healthy controls. Cannabis use disorders and alcohol and other substance use disorders (SUD) were identified through combinations of several health registers, as were the outcomes. We investigated the following outcomes: Any disorder of the digestive organs, cancers of the digestive organs, non-cancer disorders of the digestive organs, inflammatory bowel disease, disorders of the gut-brain axis, non-cancer disorders of the pancreas, non-cancer disorders of the liver, and a category of disorders of the digestive organs considered to be severe. For each analysis, people with a corresponding digestive-organ diagnosis before either the date of schizophrenia or the controls’ corresponding match-date were excluded.

**Results:**

The number of patients with schizophrenia varied between 17,718 and 22,636 in different analyses. After adjusting for other SUDs, cannabis use disorders showed protective effects in patients with schizophrenia against disorders of the gut-brain axis (HR=0.78, 95% CI 0.68–0.91, p=0.001), and serious disorders of the digestive organs (HR=0.81, 95% CI 0.69–0.96, p=0.02). Non-significant protective effects were also apparent for non-cancer disorders of the digestive organs (HR=0.93, 95% CI 0.85–1.02, p=0.11), inflammatory bowel disease (HR=0.67, 95% CI 0.42–1.08, p=0.10), and non-cancer disorders of the liver (HR=0.80, 95% CI 0.61–1.05, p=0.12). These protective effects were never shown in healthy controls, where cannabis use disorders generally were not associated in any direction with digestive-organ disorders in the fully adjusted analyses.

**Discussion:**

Cannabis use disorders may show protective effects against certain disorders of the digestive organs in patients with schizophrenia. The explanations may be causal, which would however require further explanations as to why the same effects are not seen in healthy controls. This may be due to use of antipsychotic medication interacting with cannabis, or due to higher levels of cannabis consumption in cases with schizophrenia than in controls. The effect may also be in part explained by comorbid use of tobacco, as nicotine is also known to interact with the gastrointestinal system.

